# Factor VIII administration during emicizumab prophylaxis: how much is enough?

**DOI:** 10.1016/j.rpth.2026.106952

**Published:** 2026-09-09

**Authors:** Runhui Wu

**Affiliations:** Hematology Department, Beijing Key Laboratory of Pediatric Hematology Oncology, National Key Discipline of Pediatrics (Capital Medical University), Key Laboratory of Major Diseases in Children, Ministry of Education, Beijing Children’s Hospital, National Center for Children’s Health, Capital Medical University, Beijing, China

Emicizumab is a bispecific antibody that mimics the cofactor function of activated factor VIII (FVIII) by bridging FIXa and FX, thereby establishing subcutaneous prophylaxis as a standard of care for hemophilia A with or without inhibitors [1]. Yet one question, recurring in daily practice but long unsupported by prospective evidence, has remained open: when a patient treated on emicizumab experiences a breakthrough bleed or requires surgery, how much additional FVIII should be given?

Global coagulation assays place the hemostatic activity of emicizumab at roughly that of mild hemophilia, equivalent to ∼10% to 20% FVIII activity [[2], [3], [4]]. Mechanically applying the traditional target of “correcting FVIII to 100%” therefore ignores the baseline protection emicizumab already provides and risks overcorrection—superimposing excess FVIII on an already procoagulant background, with a theoretical thrombotic risk and unnecessary factor consumption. Under correction, conversely, leaves hemostasis insufficient when higher activity is genuinely required. This “overcorrection vs undercorrection” tension is precisely what recent international consensus has flagged as unresolved [5]. The CAGUYAMA study (jRCTs051210137) was designed to answer it, with its protocol published in 2023 [6] and final results in 2026 [7].

CAGUYAMA was a multicenter, open-label, nonrandomized exploratory study conducted at 13 Japanese centers enrolling 100 patients (aged ≥4 years) with hemophilia A without inhibitors who were receiving emicizumab prophylaxis; those with breakthrough bleeds or undergoing procedures received an initial FVIII dose of 30 ± 5 U/kg, with global coagulation potential measured before and after [6,7]. Across 32 FVIII-treated events (15 breakthrough bleeds, 17 procedures) in 24 patients, FVIII raised coagulation potential from an emicizumab-supported baseline to near-normal on clot waveform analysis (median index of maximum coagulation rate 39.4%→92.1%); neutralizing emicizumab with antiemicizumab antibodies confirmed the FVIII effect was additive to emicizumab’s baseline, with a similar trend on thrombin generation and lower sensitivity on rotational thromboelastometry [7,8]. Effective hemostasis was achieved in all 32 events, with no thromboembolism or thrombotic microangiopathy [7]. The study thus provides the first prospective human evidence that FVIII adds to—rather than simply replaces—the hemostatic potential of emicizumab, and that a moderate 30 ± 5 U/kg starting dose is feasible for bleeds and many procedures.

The design of this study is innovative. By using antiemicizumab antibodies to dissect, within a single blood sample, the separate contributions of emicizumab and FVIII, CAGUYAMA converts a long-standing mechanistic inference—previously derived largely from *in vitro* and *ex vivo* models [8,9]—into quantified, prospective, multicenter clinical evidence. Clinically, the message is reassuring: 32 events, including tooth extraction, synovectomy, and even total knee arthroplasty, achieved effective hemostasis at a moderate 30 ± 5 U/kg starting dose without thrombotic complications. This supports a pragmatic shift away from reflexively correcting FVIII to 100% and toward titrating from a moderate dose to clinical response—reducing overcorrection while preserving efficacy. The work also clarifies assay roles: corrected clot waveform analysis is most sensitive to emicizumab’s baseline activity, thrombin generation gives an overall view, and rotational thromboelastometry serves as a whole-blood adjunct in high-risk perioperative settings [7,8]—complementary tools, not interchangeable ones.

These findings still have some limitations, particularly for children. Although eligibility began at age 4, the enrolled population was predominantly adult (median age at events 35 years, range 4-73), and pediatric patients and events were not separately reported [7]. The 30 ± 5 U/kg conclusion therefore lacks independent efficacy and pharmacokinetic support in children, in whom faster FVIII clearance and age-dependent recovery and half-life may yield exposure quite different from the adult-derived figures. The evidence base is also modest and exploratory: 32 events, no control dose group, no randomization—and the 2 major orthopedic procedures in fact required somewhat more than 30 ± 5 U/kg, a reminder that major surgery still warrants individualized management. Finally, the global coagulation assays central to this approach remain largely confined to specialist laboratories and are not yet standardized across centers, and all data derive from Japanese practice, so extrapolation to other populations and health systems calls for caution.

Where does this leave us? CAGUYAMA offers a valuable conceptual framework: because emicizumab already delivers baseline hemostatic activity and FVIII adds to it, replacement strategies designed for the preemicizumab era should not be applied unchanged. The baseline contribution of emicizumab should inform the choice of an initial FVIII dose rather than defaulting to conventional target dose. For children, 30 ± 5 U/kg is best read as the regimen CAGUYAMA evaluated, not a validated pediatric recommendation—supplementation should remain individualized by age, weight, bleeding severity, procedural risk, and pharmacokinetics, and established perioperative targets for major surgery should not be lowered on the strength of this study alone. It is also worth recalling that conventional one-stage activated partial thromboplastin time–based FVIII assays substantially overestimate activity in the presence of emicizumab, so validated assays are essential whenever FVIII monitoring is required [10,11]. Future prospective work should define age-specific FVIII pharmacokinetics, characterize efficacy and safety across bleeding and surgical types, and test whether global coagulation assays can genuinely guide individualized dosing.
